# Study on CMC-Based Suppressant for Coal Dust and Spontaneous Combustion Control

**DOI:** 10.3390/polym18172043

**Published:** 2026-08-23

**Authors:** Jianguo Wang, Tianle Jia, Zhenzhen Zhang, Xinni He

**Affiliations:** College of Safety Science and Engineering, Xi’an University of Science and Technology, Xi’an 710054, China; j537557002@163.com (T.J.); 13834846998@163.com (X.H.)

**Keywords:** flame-retardant dust suppressant, carboxymethyl cellulose, polycarbodiimide, coal spontaneous combustion, dust suppression, apparent activation energy

## Abstract

Underground coal mining faces coupled hazards from respirable coal dust and spontaneous coal combustion. This study developed a dual-function flame-retardant dust suppressant comprising carboxymethyl cellulose (CMC), polycarbodiimide (PCDI), ammonium polyphosphate (APP), and zinc borate (ZB). A four-factor, three-level orthogonal design was used to screen formulations by penetration depth, followed by rheological, water-scour, simulated-roadway, temperature-programmed oxidation, contact-angle, Fourier Transform Infrared Spectroscopy (FTIR), and Scanning Electron Microscope (SEM) analyses. CMC and PCDI significantly affected penetration, whereas APP and ZB showed no significant effects within the tested ranges. The selected formulation (1% CMC, 12% APP, 3.5% ZB, and 1.5% PCDI) showed stable viscosity development and the lowest mass loss under repeated water scour. In simulated-roadway tests, the stock solution achieved an average dust-suppression efficiency of 59.7%. At 170 °C, a 10% treatment reduced CO release by 40.0% and increased the mean apparent activation energy of coal oxidation by 29.73%. Rapid wetting, intermolecular interactions, and formation of a continuous porous crosslinked film supported dust consolidation and oxidation inhibition. The developed material therefore offers a potential integrated approach for controlling coal dust and spontaneous combustion risks in underground mines.

## 1. Introduction

Coal remains a major source of industrial energy and electricity in leading coal-producing countries, including China, Australia, the United States, and India [1,2,3]. However, underground mining operations face two persistent hazards: coal dust and spontaneous coal combustion. Mechanical comminution during excavation, tunneling, and coal handling generates large quantities of respirable dust, which can cause pneumoconiosis [4,5] and form explosive clouds under turbulent airflow [6]. In parallel, fractured coal rich in reactive functional groups [7,8] undergoes low-temperature oxidation in the presence of oxygen, releasing CO, hydrocarbons, and heat [9]. Continued heat accumulation can ultimately initiate underground fires [10], obstruct roadways, damage equipment, and waste coal resources. These hazards can reinforce one another: fine coal particles provide a larger specific surface area for coal–oxygen reactions, while oxidation-generated heat promotes dust dispersion and increases explosion risk. Their coupling therefore complicates underground hazard control.

Conventional mine-safety strategies treat dust suppression and fire prevention as separate systems, each with important limitations. Water spraying or injection provides only short-lived wetting and weak consolidation [11]. Hygroscopic inorganic salts, such as calcium chloride and magnesium chloride, release corrosive ions that can damage underground electromechanical equipment and contaminate mine water [12]. Polymer film-forming dust suppressants improve consolidation but generally lack flame-retardant components and therefore do not inhibit the low-temperature oxidation of coal [13].

Similarly, methods for controlling coal spontaneous combustion, including inert-gas injection, grouting, and gel sealing [14,15], require independent infrastructure, are costly, and often penetrate loose coal poorly. Conventional phosphorus- and boron-based inhibitors suppress oxidation but do not immobilize airborne dust, so additional dust-control operations are required. Previous work developed a nano-SiO_2_-modified polyacrylic acid suppressant based on synergistic N-P-Si effects [16]. Building on these efforts, the present study sought to integrate dust consolidation and coal spontaneous combustion inhibition in a single CMC-based material.

Polymer composites have attracted increasing interest for integrated mine-hazard control. Carboxymethyl cellulose (CMC), a water-soluble derivative of natural polysaccharides, exhibits favorable film-forming and adhesive properties and is biodegradable [17,18]. Its abundant hydroxyl and carboxyl groups promote interfacial bonding with coal, making CMC a suitable matrix for dust suppressants [19]. Crosslinking agents can improve the mechanical strength and water resistance of CMC films by creating three-dimensional networks [20]. Polycarbodiimide (PCDI) reacts covalently with carboxyl groups on CMC chains, thereby modifying solution rheology and penetration and improving the durability of consolidated dust layers [21,22,23]. Ammonium polyphosphate (APP), an intumescent phosphorus-based flame retardant, decomposes to form a phosphoric-acid-rich barrier that restricts oxygen transport and quenches oxidative radicals [24,25]. Zinc borate (ZB) can act synergistically by absorbing heat and releasing inert gases at low to intermediate temperatures [26,27]. Nevertheless, most studies have focused on either single-function CMC dust suppressants [28,29] or independent phosphorus- or boron-based coal inhibitors [30]. There are few studies on the dual-effect inhibitors of flame retardant and dust suppression. Some scholars have studied the preparation of dual inhibitors based on the synergistic effect of nitrogen, phosphorus and silicon [16], but few studies have combined CMC, PCDI, APP and ZB into integrated inhibitors. The relationship between permeability, corrosion resistance, dust consolidation and oxidation inhibition is still unclear. Systematic evaluations are also limited, including simulated road testing, temperature programmed oxidation, and multi-scale characterization.

The component selection and concentration design of this study are based on the following synergistic expectations: CMC is a natural polysaccharide derivative. The hydroxyl and carboxyl groups on the molecular chain of CMC can form hydrogen bonds with the polar functional groups on the surface of coal and act as a film-forming matrix to provide carriers for other active components. However, the pure CMC membrane has poor water resistance. Therefore, PCDI is introduced as a crosslinking agent. The carbodiimide group of PCDI can be covalently crosslinked with the carboxyl group of CMC to form a three-dimensional network structure, which not only improves the mechanical strength and erosion resistance of the membrane layer but also balances the permeability and consolidation performance by regulating the crosslinking density. As an intumescent flame retardant, APP is decomposed by heat to form a polyphosphate layer covering the surface of coal, but it easily produces a ‘candle-core effect’ when used alone. Therefore, the compound ZB-ZB decomposes and absorbs heat at 200~300 °C and releases water vapor to dilute oxygen. At the same time, it reacts with the phosphoric acid produced by APP decomposition to form glassy boron phosphate, which can improve the compactness of the carbon layer. The crosslinking efficiency of PCDI is matched with the carboxyl content of CMC. The approximate concentration range was determined by laboratory pre-experiment preparation. The four components are expected to achieve the dual functions of dust consolidation and oxidation inhibition through the multi-scale synergy of ‘physical film formation-chemical crosslinking-gas phase flame retardant-condensed phase carbonization’.

Accordingly, this study developed a dual-function flame-retardant dust suppressant using CMC as the film-forming matrix, APP and ZB as synergistic flame retardants, and PCDI as the crosslinker. A four-factor orthogonal design was used to screen formulations based on penetration depth. Rheological behavior and water-scour resistance were then evaluated to select a formulation for further study. Simulated-roadway experiments quantified dust-suppression efficiency at different solution concentrations, whereas temperature-programmed oxidation experiments assessed CO evolution and apparent activation energy. Contact-angle measurements, FTIR, and SEM were used to clarify the wetting, consolidation, and oxidation-inhibition mechanisms. This work provides a basis for the integrated control of coal dust and spontaneous combustion hazards in underground mines.

## 2. Materials and Methods

### 2.1. Materials

The materials used in this study are listed in Table 1. All chemical reagents were of analytical grade.

### 2.2. Penetration-Depth Measurement and Preliminary Formulation Screening

Penetration depth is a key performance parameter because it determines how effectively the suppressant infiltrates loose coal in underground applications. A four-factor, three-level orthogonal design (Table 2) was therefore used to prepare nine formulations. Penetration depth was measured for each formulation to identify candidates for further testing.

Coal particles of different sizes were weighed and thoroughly mixed to reproduce the particle-size distribution and packing state of a coal seam. The mixture was loaded into a vertical column whose base was sealed with gauze. A 100 mL aliquot of each suppressant formulation was poured slowly onto the top of the column. After 30 min of standing, the penetration depth within the coal bed was measured and recorded.

### 2.3. Basic Performance Tests and Formulation Optimization

An NDJ-1 rotational viscometer was used to monitor viscosity over time and evaluate the effects of formulation components on the rheological behavior of the suppressant.

Four portions of loose pulverized coal with the same particle-size distribution were prepared, and each candidate formulation was sprayed onto a separate portion. Simulated rainfall was applied five times over 2 days, with each event lasting 30 min. The mass-loss rate was calculated to evaluate resistance to water scour.

Based on the penetration test and the application-performance tests, one formulation was selected for the subsequent experiments.

To further verify the reliability of the significance analysis, post hoc power analysis was conducted using G*Power 3.1. For the current orthogonal design (df = 2, F = 5.281 for PCDI, α = 0.05), the achieved power (1–β) was 0.82, which exceeds the conventional threshold of 0.8, indicating sufficient statistical power to detect the effects of PCDI and CMC on penetration depth despite the small per-group sample size.

### 2.4. Dust-Suppression Performance Test

**Figure 1:**
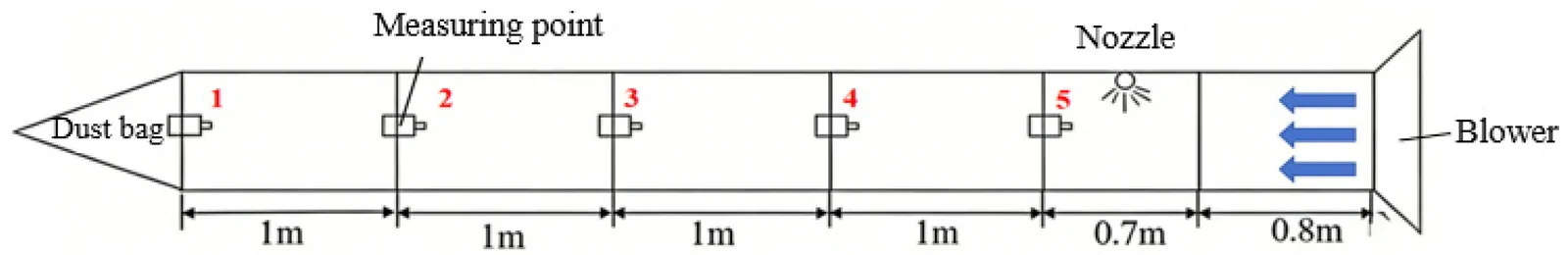
Layout of the measurement points and nozzle.

Dust-suppression performance was evaluated in a custom simulated roadway constructed from 1 cm thick acrylic sheets. The roadway measured 0.6 m × 0.6 m × 5.5 m; both ends were open, whereas the side walls were sealed. Dust-concentration monitors were positioned at 1 m intervals along the roadway (Figure 1). Pulverized coal with a particle size of 0–0.9 mm was used as the dust source, and its analytical characteristics are listed in Table 3. The nozzle injection pressure was maintained at 0.5 Mpa.

### 2.5. Flame-Retardancy Test

Temperature-programmed oxidation experiments were conducted to simulate the heating conditions associated with the low-temperature oxidation and spontaneous combustion of coal. Three groups of samples were set up: raw coal was the control group; the experimental groups were coal samples with 5% and 10% flame retardant and dust suppressant materials, respectively. The test temperature range is 30~170 °C, the heating rate is 1 °C/min, and the gas sampling is carried out once every 10 °C increase. When each gas is sampled, 5 tubes of gas are taken, and the first 3 tubes of gas are naturally discharged. The fourth tube of gas is used for subsequent testing, and the fifth tube of gas is used for standby. The sampling gas was injected into the gas chromatograph for the determination of composition and content, and the relevant data were recorded.

### 2.6. Characterization

FTIR spectra were acquired using a Thermo Fisher Scientific Nicolet iS20 spectrometer, manufactured in the Waltham, Massachusetts, United States over 4000–400 cm^−1^ with 32 scans per sample. The flame-retardant material and coal samples were analyzed separately.

Microstructural characterization was performed using a ZEISS Sigma 360 (Carl Zeiss Microscopy GmbH, Jena, Germany) scanning electron microscope operated at an accelerating voltage of 15 kV and a working distance of 5–10 mm.

The contact angle of the suppressant on pressed coal pellets was measured using a LAUDA Scientific LSA100S optical (LAUDA Scientific GmbH, Lauda-Königshofen, Germany) contact-angle instrument.

## 3. Results and Discussion

### 3.1. Effects of Formulation Variables on Penetration Depth

The measured penetration depths are summarized in Table 4.

**Figure 2:**
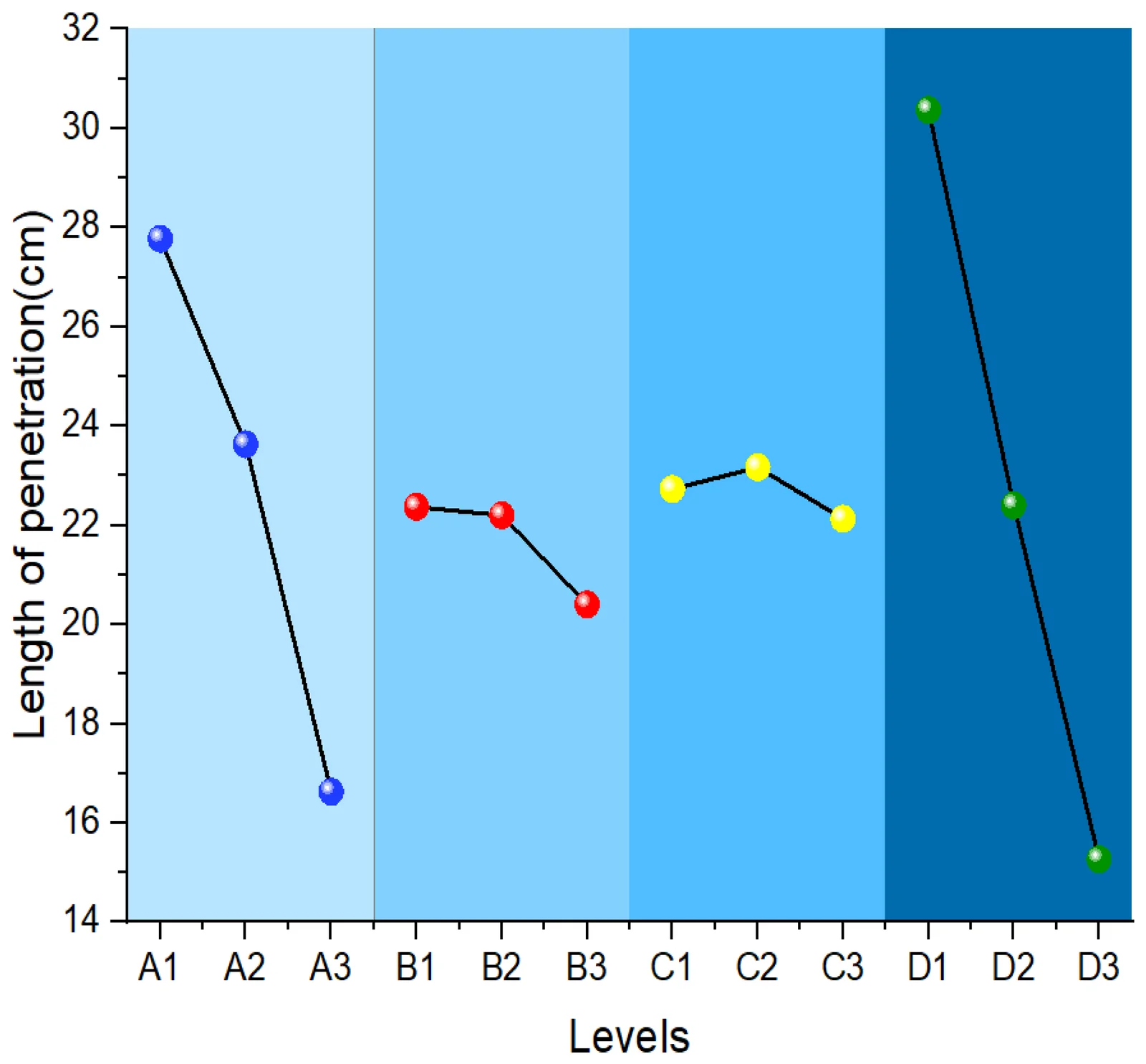
The influence of each factor level on the penetration depth.

The effects of factor levels on penetration depth are shown in Figure 2, and the corresponding significance analysis is presented in Table 5.

The regression model yielded R^2^ = 0.956 (*p* < 0.05), adjusted R^2^ = 0.932; predicted R^2^; Lack-of-Fit (F-value) = 2.14; Lack-of-Fit (*p*-value) = 0.172, indicating good agreement with the experimental data. CMC (F = 1.644, *p* = 0.043) and PCDI (F = 5.281, *p* = 0.025) significantly affected penetration depth. By contrast, APP (F = 0.016, *p* = 0.384) and ZB (F = 0.009, *p* = 0.591) had no significant effects within the tested ranges.

The relative influence of the four factors followed the order PCDI > CMC > APP > ZB.

PCDI concentration controls the degree of crosslinking and therefore affects solution permeability. CMC regulates apparent viscosity, rheological behavior, and interfacial wettability, thereby balancing penetration resistance and penetration rate. Its interactions with PCDI also contribute to the formation of a gradient crosslinked network. Together, PCDI and CMC govern penetration through coupled effects on viscosity, mass transfer, interfacial behavior, and microstructure.

Accordingly, jointly adjusting the PCDI and CMC contents provides a practical means of optimizing penetration into porous coal.

Four candidate formulations were selected for further testing: 1% CMC + 8% APP + 2% ZB + 0.5% PCDI; 1% CMC + 12% APP + 3.5% ZB + 1.5% PCDI; 1.5% CMC + 16% APP + 3.5% ZB + 0.5% PCDI; and 2% CMC + 12% APP + 5% ZB + 0.5% PCDI.

### 3.2. Rheological Behavior

**Figure 3:**
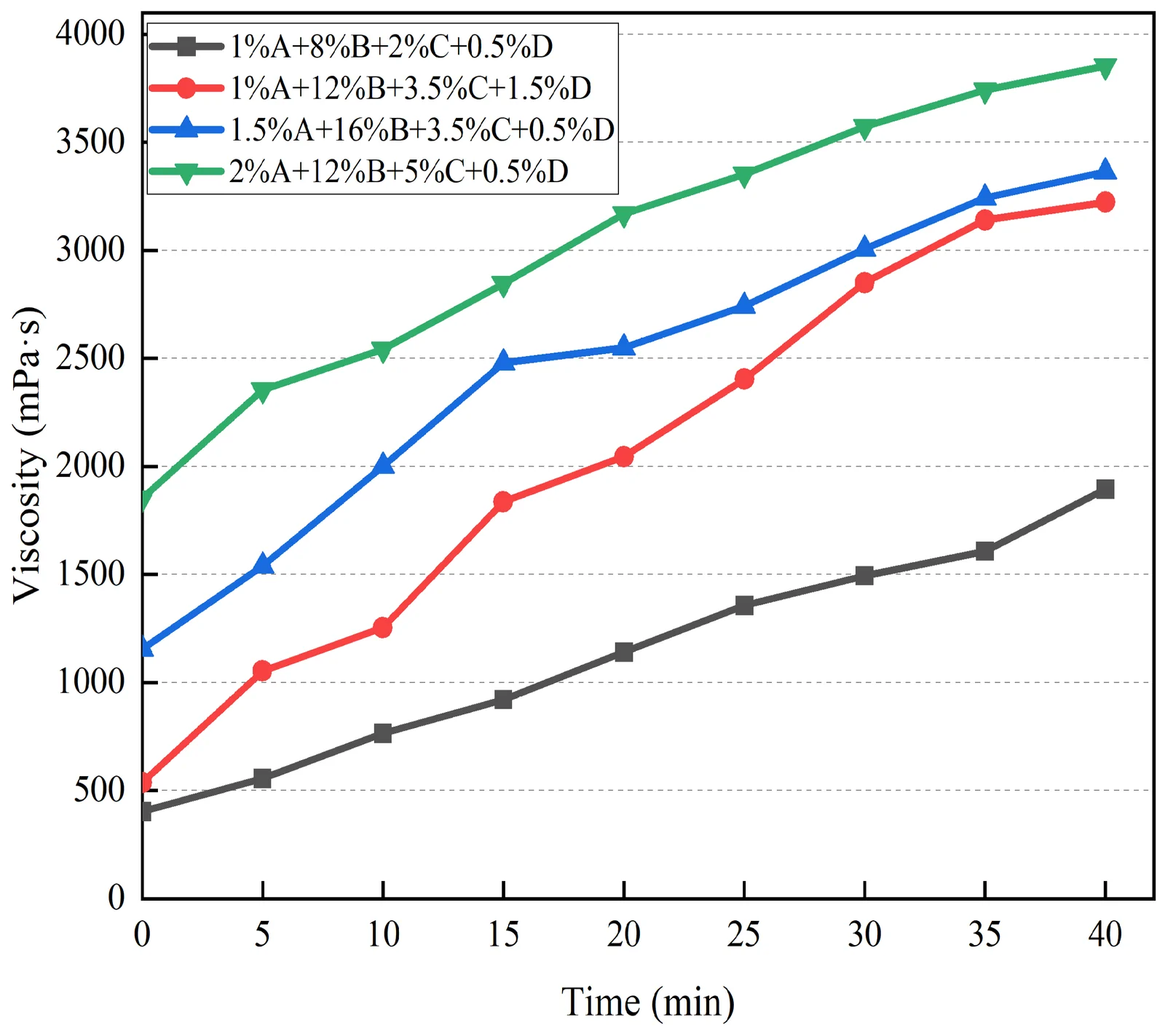
Viscosity as a function of time.

Figure 3 shows the viscosity–time curves of the four candidate formulations.

Viscosity increased over time for all four formulations. This behavior indicates progressive crosslinking among the components, which produced a denser network, reduced system fluidity, and increased viscosity.

Viscosity increased over time for all four formulations, which was primarily attributed to the progressive covalent crosslinking between the carbodiimide groups (-N=C=N-) of PCDI and the carboxyl groups of CMC, forming a denser three-dimensional network that reduced system fluidity. However, viscosity evolution was also influenced by multiple coupled factors: the concentration of CMC polymer chains, which determined the initial entanglement density of the system; intermolecular hydrogen bonding between CMC hydroxyl/carboxyl groups and APP/ZB surface polar sites, which enhanced chain aggregation; the dispersion state of APP and ZB particles, where higher inorganic filler loading increased solution viscosity through particle–polymer interactions and hydrodynamic resistance. The differences in viscosity–time curves among formulations further reflected the combined effects of these factors: higher CMC content increased the initial polymer concentration and potential crosslinking sites, while higher PCDI content accelerated the crosslinking reaction rate and final network compactness.

### 3.3. Resistance to Water Scour

For the water-scour test, sample mass was recorded before and after each event, and the mass-loss rate was calculated using Equation (1).

(1) $$Q_{R}=\frac{m_{0}-m_{1}}{m_{0}}\times 100\%$$

Here, Q_R_ is the mass-loss rate (%), m_0_ is the mass after suppressant application (g), and m_1_ is the mass after the first, second, third, fourth, or fifth scour event (g).

**Figure 4:**
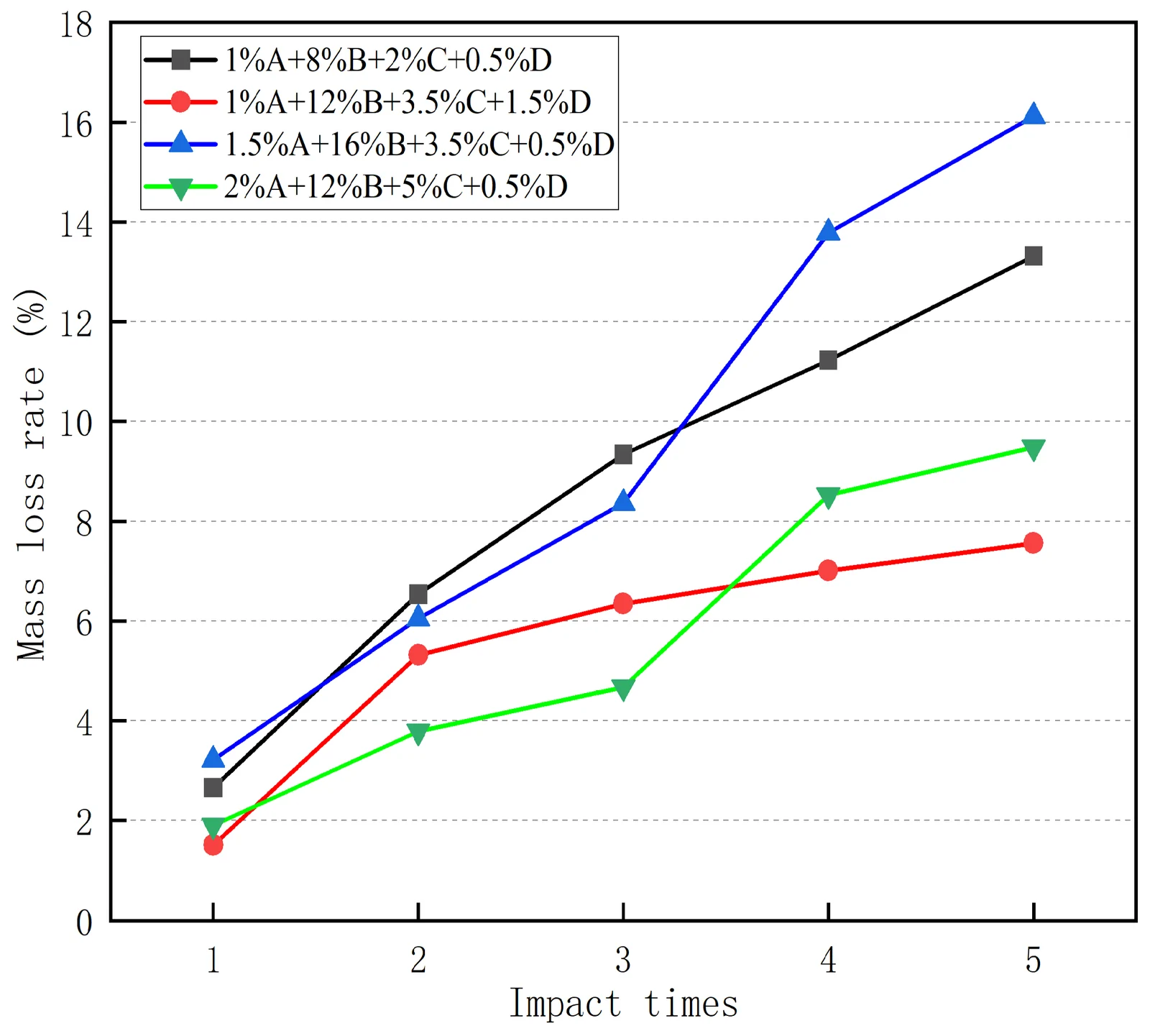
Mass-loss rate during repeated water scour.

Figure 4 shows the mass-loss rates of the four formulations during repeated water scour.

All formulations showed their lowest mass-loss rate during the first scour event, indicating that the initially consolidated layers withstood the first water impact. Mass loss increased from the second event onward, and differences among formulations became more pronounced. The formulations containing 1% CMC + 8% APP + 2% ZB + 0.5% PCDI and 1.5% CMC + 16% APP + 3.5% ZB + 0.5% PCDI showed comparatively rapid cumulative damage and particle detachment. In contrast, the formulation containing 1% CMC + 12% APP + 3.5% ZB + 1.5% PCDI exhibited the most stable mass-loss profile, indicating greater structural stability under repeated water impact.

### 3.4. Simulated-Roadway Test

**Figure 5:**
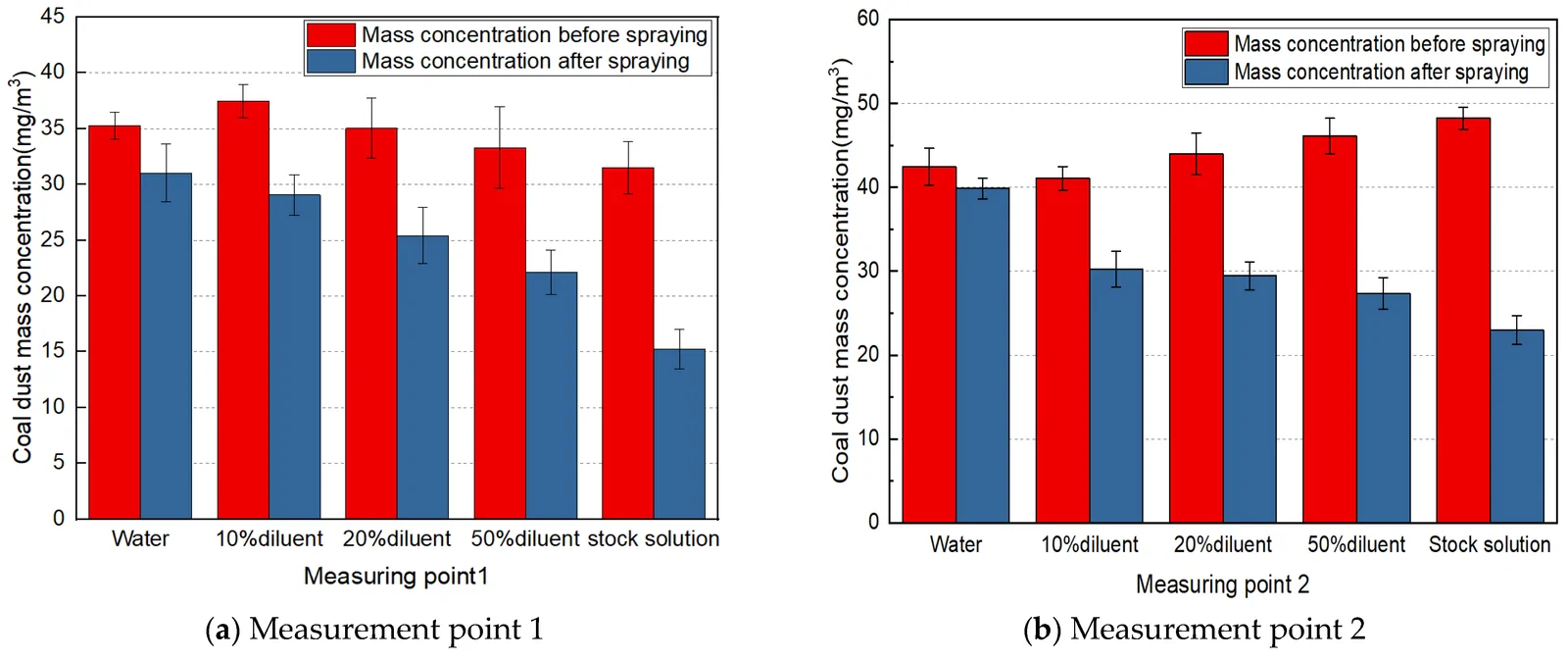

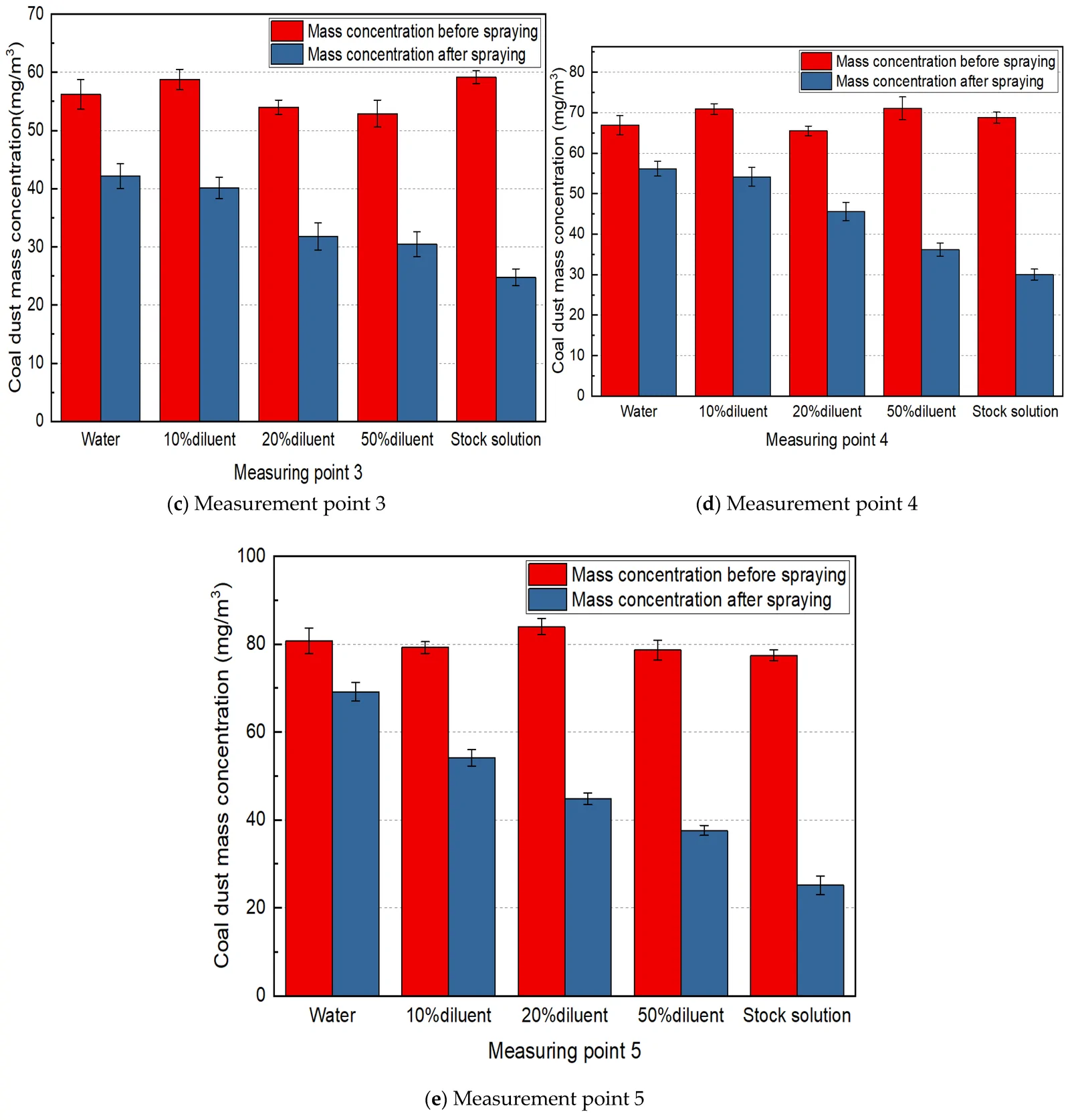
Coal-dust mass concentrations along the simulated roadway.

Figure 5 shows the coal-dust mass concentration at each measurement point in the simulated roadway.

For each measurement point, dust-suppression efficiency was calculated from the dust concentrations before and after spraying using Equation (2):

(2) $$\eta =\frac{C_{b}-C_{a}}{C_{b}}\times 100\%$$

where η is the dust-suppression efficiency (%), $C_{b}$ is the coal-dust concentration before spraying (mg/m^3^), and $C_{a}$ is the coal-dust concentration after spraying (mg/m^3^).

**Figure 6:**
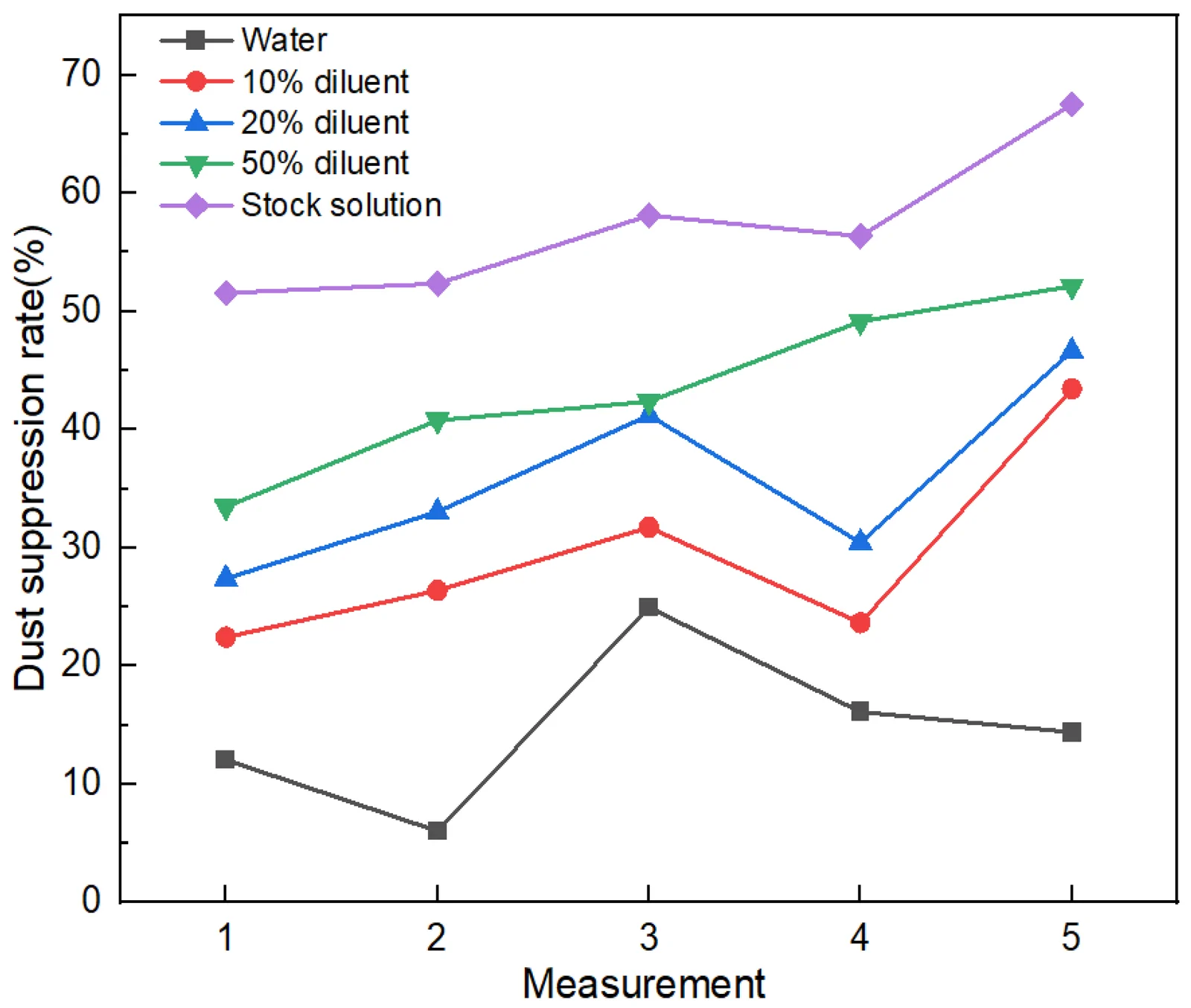
Dust-suppression efficiency at different measurement points.

Figure 6 compares the dust-suppression efficiencies of water and the suppressant at different solution concentrations and measurement points.

Dust-suppression efficiency increased consistently with suppressant concentration and remained relatively stable along the roadway (Figure 6). Water produced the poorest performance, with efficiencies of only 5–25%. Increasing the suppressant concentration progressively improved dust control. The stock solution achieved the highest efficiency, with the best result recorded at measurement point 5.

This concentration dependence reflects changes in the amount of active binder, viscosity, film-forming capacity, and adhesive strength. Water wets the particles but lacks the binding and film-forming components required for persistent agglomeration, so particles can be readily re-entrained by airflow. The stock solution contained the highest concentration of active components and therefore provided the strongest agglomeration and film formation. Its average dust-suppression efficiency was 59.7%, and the efficiency at measurement point 5 reached 67.5%.

### 3.5. Temperature-Programmed Oxidation Experiments

Temperature-programmed oxidation experiments were used to evaluate flame-retardant performance under conditions simulating the heating of coal during low-temperature oxidation. CO and other gases released from raw coal and from coal treated with different amounts of suppressant were measured as functions of temperature. The resulting data were used to determine characteristic temperatures and calculate apparent activation energies.

**Figure 7:**
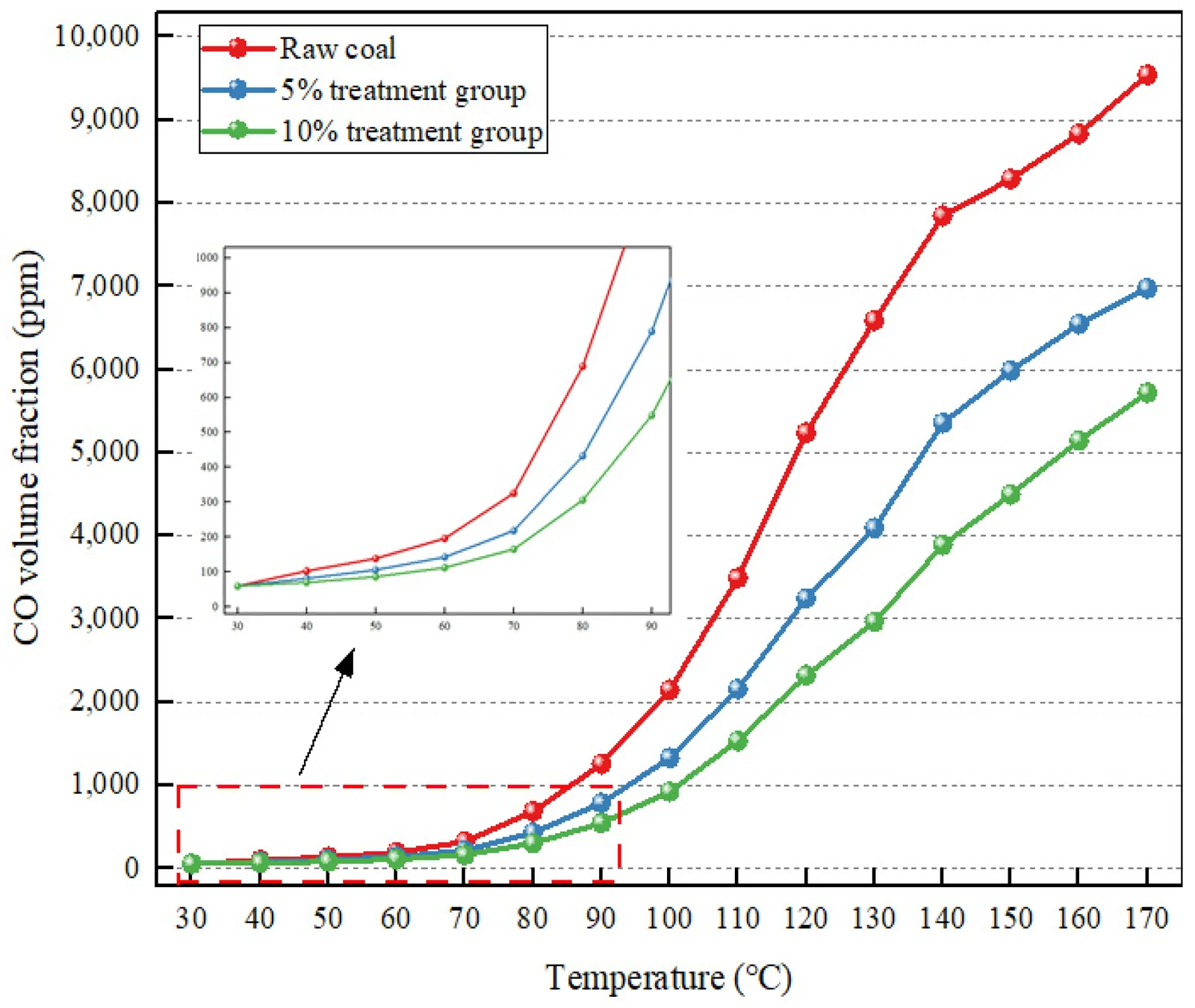
CO volume fraction as a function of temperature.

Figure 7 shows the CO volume fractions for raw coal and the suppressant-treated groups.

CO release decreased as the suppressant dosage increased. At 170 °C, the raw-coal group produced 9542.3 ppm CO, compared with 6975.5 ppm for the 5% treatment group and 5721.3 ppm for the 10% treatment group. These values correspond to reductions of approximately 26.9% and 40.0%, respectively. Thus, the suppressant delayed low-temperature coal oxidation, and the inhibitory effect increased with dosage.

A plausible inhibition mechanism involves complementary physical and chemical effects. The CMC matrix and PCDI crosslinker enhance adhesion and form a dense film on the coal surface, thereby restricting oxygen access to reactive sites. Meanwhile, APP and ZB act synergistically: APP-derived phosphorus-containing products form a surface barrier and suppress radical-chain reactions, whereas gas released during thermal decomposition further limits oxygen transport. Together, these effects reduce CO formation.

To quantify inhibition, the CO inhibition rate was calculated from the difference between the CO volume fractions of raw and treated coal using Equation (3):

(3) $$\eta =\frac{V_{rc}-V_{t}}{V_{Tt}}\times 100\%$$

where η is the CO inhibition rate (%), V_rc_ is the CO volume fraction of raw coal (ppm), and V_t_ is the CO volume fraction of the corresponding treated coal (ppm).

**Figure 8:**
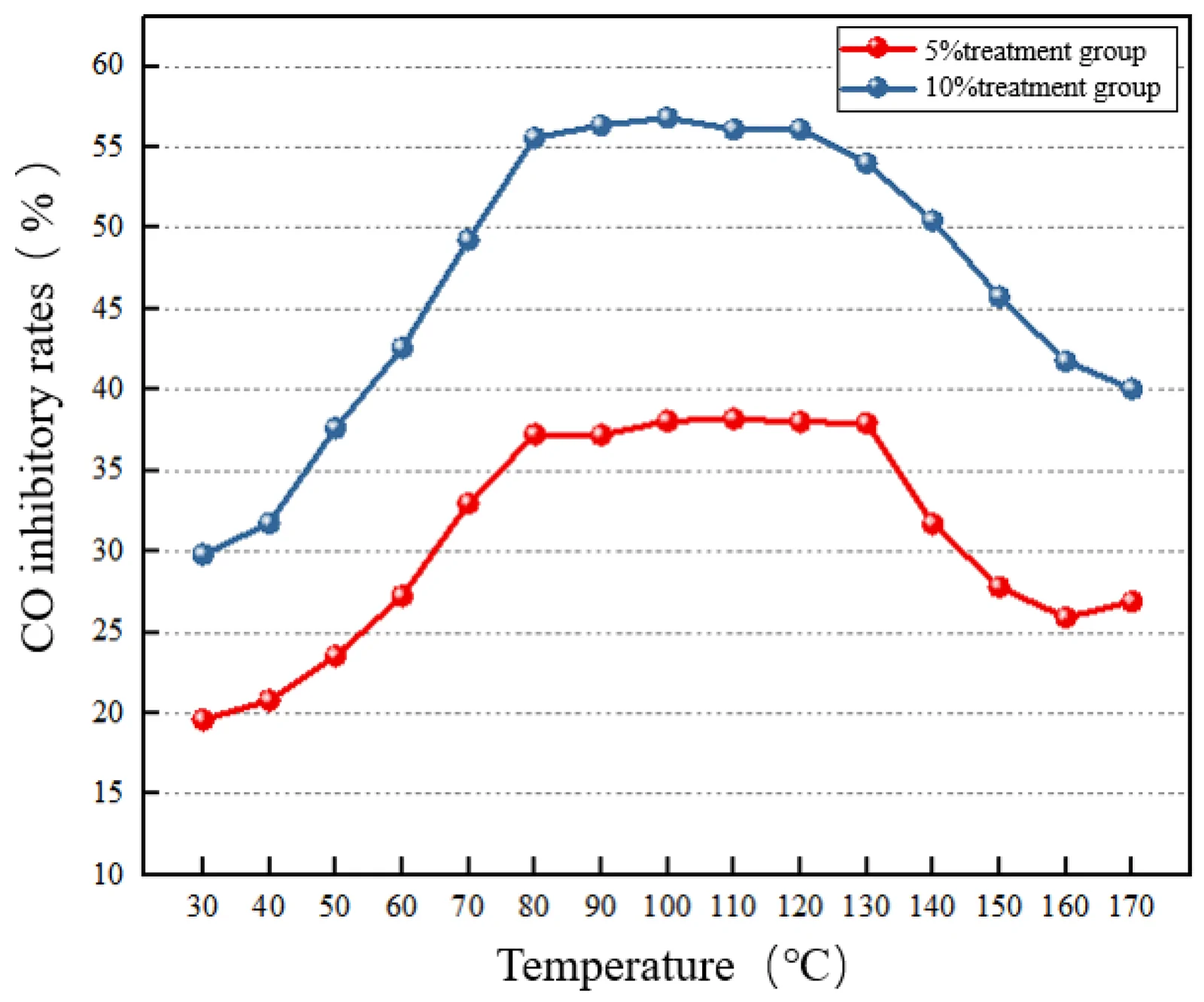
CO inhibition rate as a function of temperature.

The CO inhibition rates calculated using Equation (3) are plotted as functions of temperature in Figure 8.

Between 30 and 70 °C, the CO inhibition rate increased with temperature in both treatment groups, reaching approximately 32.97% for the 5% group and 49.26% for the 10% group. As coal oxidation became more active, the flame-retardant components also became increasingly effective. APP- and ZB-derived products covered the coal surface and suppressed oxidative radical-chain reactions, while the crosslinked matrix improved adhesion and restricted oxygen access. These combined effects account for the increasing inhibition rate in this temperature range.

Above 80 °C, the CO inhibition rates gradually decreased before stabilizing at approximately 25% for the 5% group and 40% for the 10% group. Sustained heating may partially disrupt the crosslinked protective layer and reduce its compactness. Nevertheless, inert gases and condensed-phase products generated from APP and ZB continue to restrict oxygen transport and oxidation, allowing the inhibition rate to remain stable rather than decline continuously.

Apparent activation energy provides a quantitative measure of the temperature-dependent reactivity of coal during spontaneous combustion. Combining the reaction-rate expression with the Arrhenius equation yields an activation-energy relationship based on CO evolution:

(4) $$V_{O_{2}}=V_{CO}/m=Ac_{O_{2}}^{n}\exp(E/RT_{i})$$

where the terms represent the stoichiometric coefficient of CO, the pre-exponential factor, the oxygen integral (ppm), the reaction order, the apparent activation energy E (J/mol), the molar gas constant R (8.314 J/(mol·K)), and the absolute temperature T_i_ (K), respectively.

The derivation assumes a uniform temperature distribution within the sample chamber, unidirectional gas flow, and constant sample mass during the experiment. For an infinitesimal axial segment dx of the sample chamber, the CO generation-rate equation can be written as follows:

(5) $$SV_{CO}dx=kV_{g}dc$$

where k is the unit-conversion coefficient (22.4 × 10^9^), V_g_ is the gas-flow velocity (m/s), and c is the CO volume fraction (ppm).

Substituting Equation (4) into Equation (5) gives the following:

(6) $$SmAc_{O_{2}}^{n}\exp(-E/RT_{i})dx=kV_{g}dc$$

Integrating both sides yields the following:

(7) $$SmAc_{O_{2}}^{n}L\exp(E/RT_{i})=kV_{g}c_{t}$$

where c_t_ is the CO volume fraction of the corresponding coal sample (ppm).

Taking the natural logarithm of both sides gives the following:

(8) $$\ln c_{t}=\frac{E}{RT_{i}}+\ln(\frac{mSALc_{O_{2}}^{n}}{kV_{g}})$$

At a constant gas-flow rate, the grouped flow-related term can be treated as a constant, giving a linear relationship between ln c_t_ and 1/T_i_. Accordingly, plotting ln c_t_ against 1/T_i_ and fitting a straight line allows the apparent activation energy E to be calculated from the slope.

**Figure 9:**
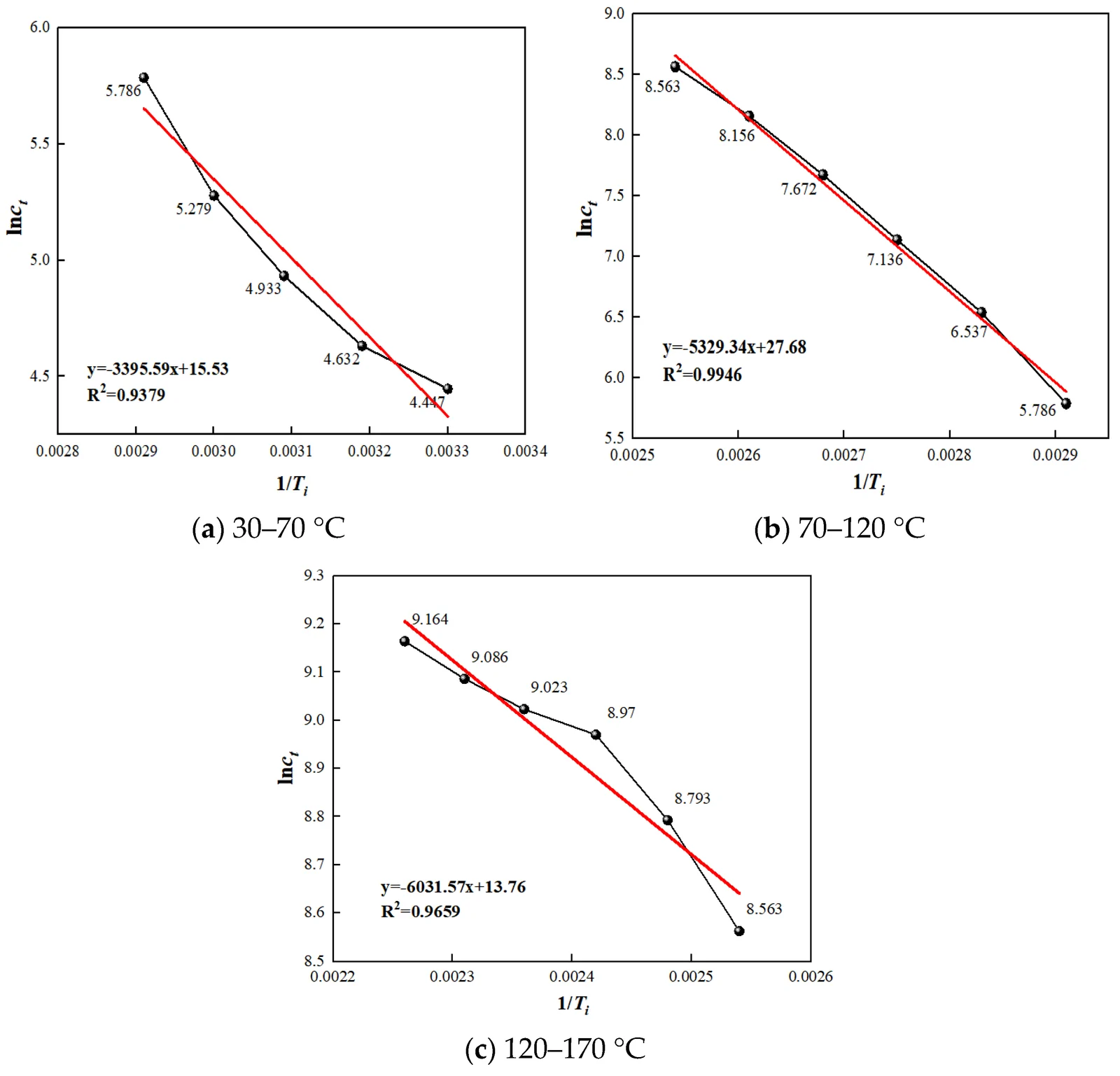
Linear fits of ln ct versus 1/Ti for raw coal in the three temperature intervals.

**Figure 10:**
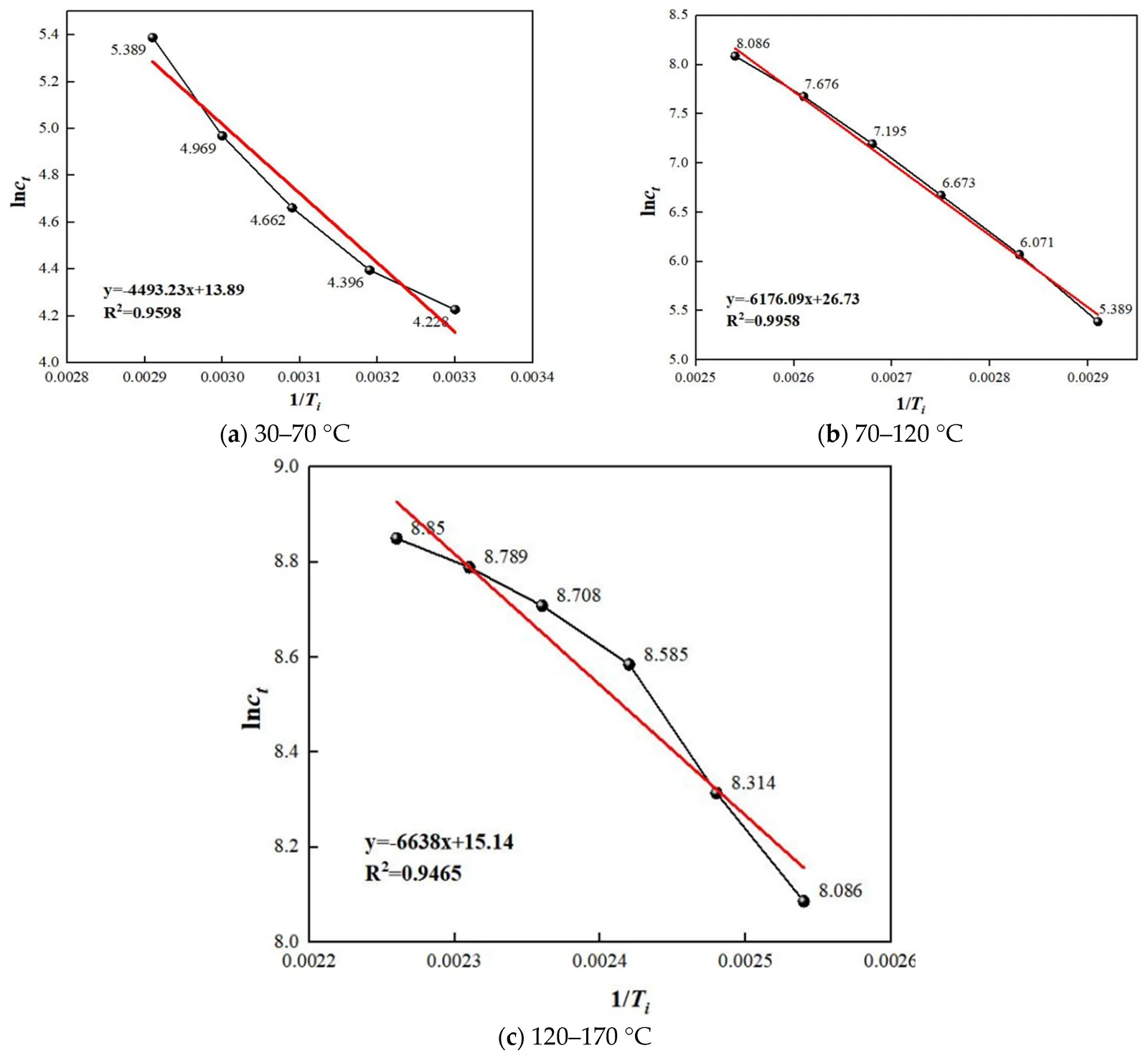
Linear fits of ln ct versus 1/Ti for coal treated with 5% suppressant.

**Figure 11:**
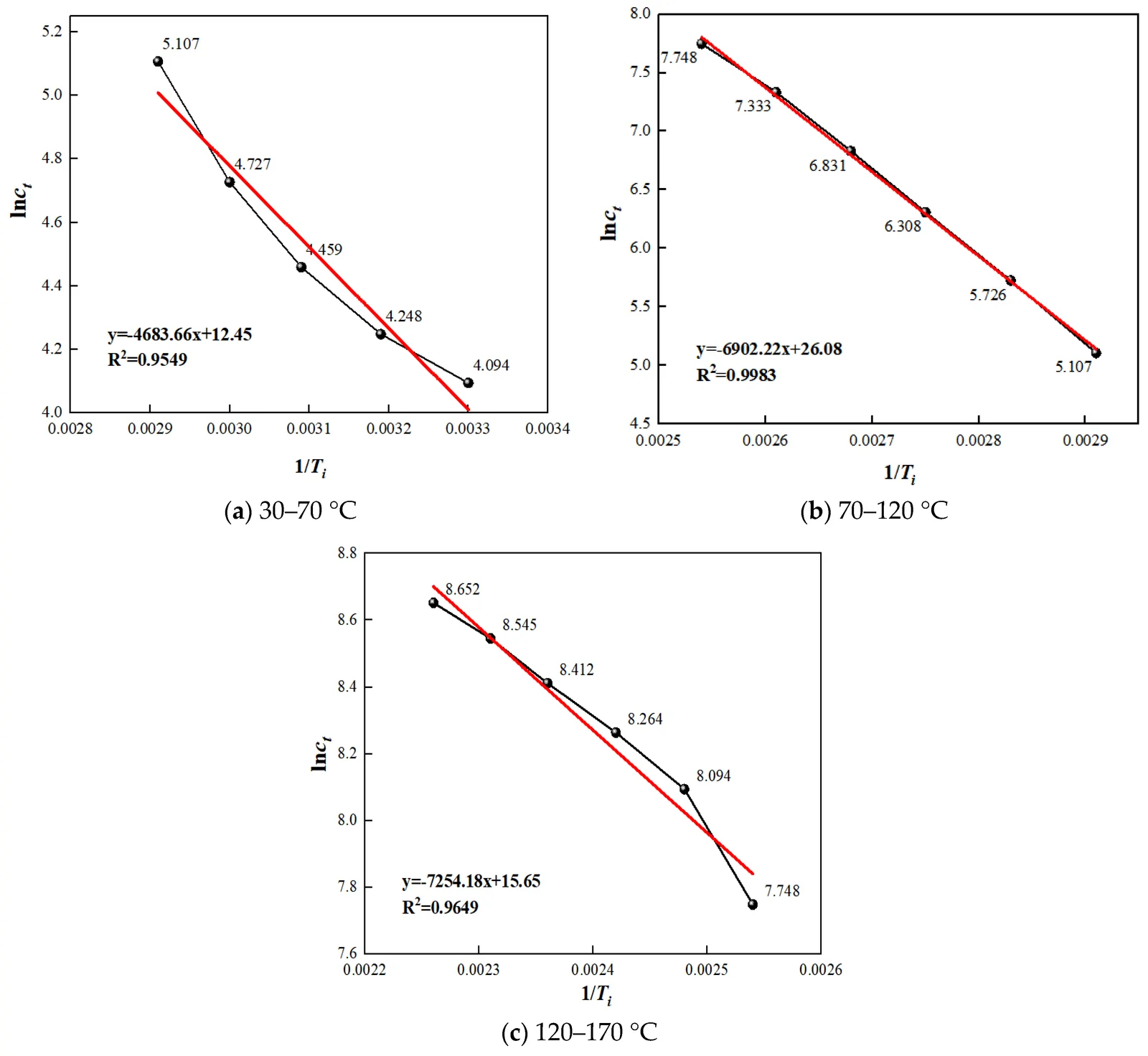
Linear fits of ln ct versus 1/Ti for coal treated with 10% suppressant.

Changes in CO concentration and formation rate identified a critical temperature of 70 °C and a dry-cracking temperature of 120 °C. Because the oxidation rate changed markedly at these temperatures, apparent activation energies were calculated for three intervals: the pre-critical stage (30–70 °C), the critical-to-dry-cracking stage (70–120 °C), and the post-dry-cracking stage (120–170 °C). The corresponding relationships between ln c_t_ and 1/T_i_ are shown for raw coal in Figure 9, the 5% treatment group in Figure 10, and the 10% treatment group in Figure 11.

The coefficients of determination (R^2^) exceeded 0.9 for all groups and temperature intervals, indicating that the linear models adequately represented the data. Apparent activation energies were calculated from the fitted slopes and are listed in Table 6.

As shown in Table 6, the apparent activation energy increased across successive temperature intervals, although the magnitude of the increase became smaller. This stage-dependent behavior may reflect the progressive consumption of more reactive sites and a shift toward reactions requiring greater bond-cleavage energy. The smaller increment at higher temperature may result from depletion of the functional groups available for oxidation.

The treated samples had higher apparent activation energies than raw coal in all temperature intervals. The mean increases were 19.59% for the 5% treatment group and 29.73% for the 10% treatment group. These results indicate that the suppressant increased the energy barrier for coal–oxygen reactions and that inhibition strengthened with increasing dosage.

### 3.6. Contact-Angle Measurements

The wettability and spreading behavior of the optimized suppressant on pressed coal were quantified using a LAUDA Scientific LSA100S made in Germany, optical contact-angle instrument. These measurements were used to clarify how the solution limits the dispersion of coal dust [31].

**Figure 12:**
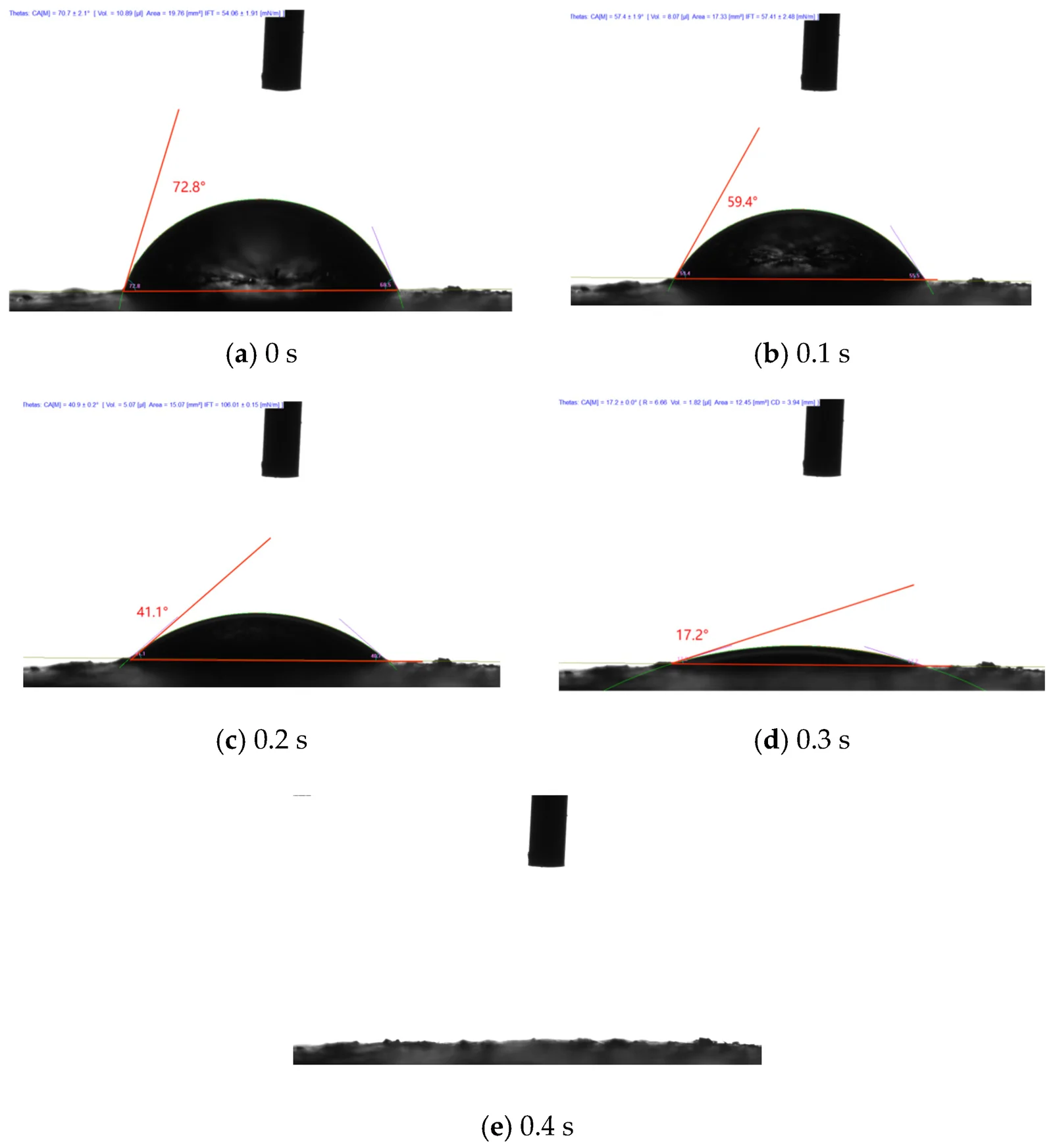
Evolution of droplet morphology and contact angle during wetting.

Coal-dust wetting can be divided into three stages: initial contact with the particles, spreading over the particle surfaces, and near-complete wetting. The corresponding droplet morphologies and contact-angle changes are shown in Figure 12.

The time-dependent contact angles and droplet states are summarized in Table 7.

The suppressant exhibited rapid dynamic wetting on the coal surface (Table 7). Within 300 ms of deposition, the contact angle decreased from 72.8° to 17.2°, a reduction of 55.6°, after which the droplet penetrated the coal. This rapid decrease confirms that the solution markedly improved surface wettability and promoted spreading.

The rapid wetting behavior can be attributed to dynamic adsorption of active components at the coal–water interface and the resulting reduction in interfacial tension. Within 300 ms, suppressant molecules migrate to the interface, drive droplet spreading, and promote penetration into pores within the dust layer. A stable hydrophilic film then forms on the coal surface. This combination of rapid spreading, deep wetting, and film formation allows the suppressant to envelop and agglomerate dust particles, thereby limiting their entrainment and dispersion.

### 3.7. FTIR Analysis

**Figure 13:**
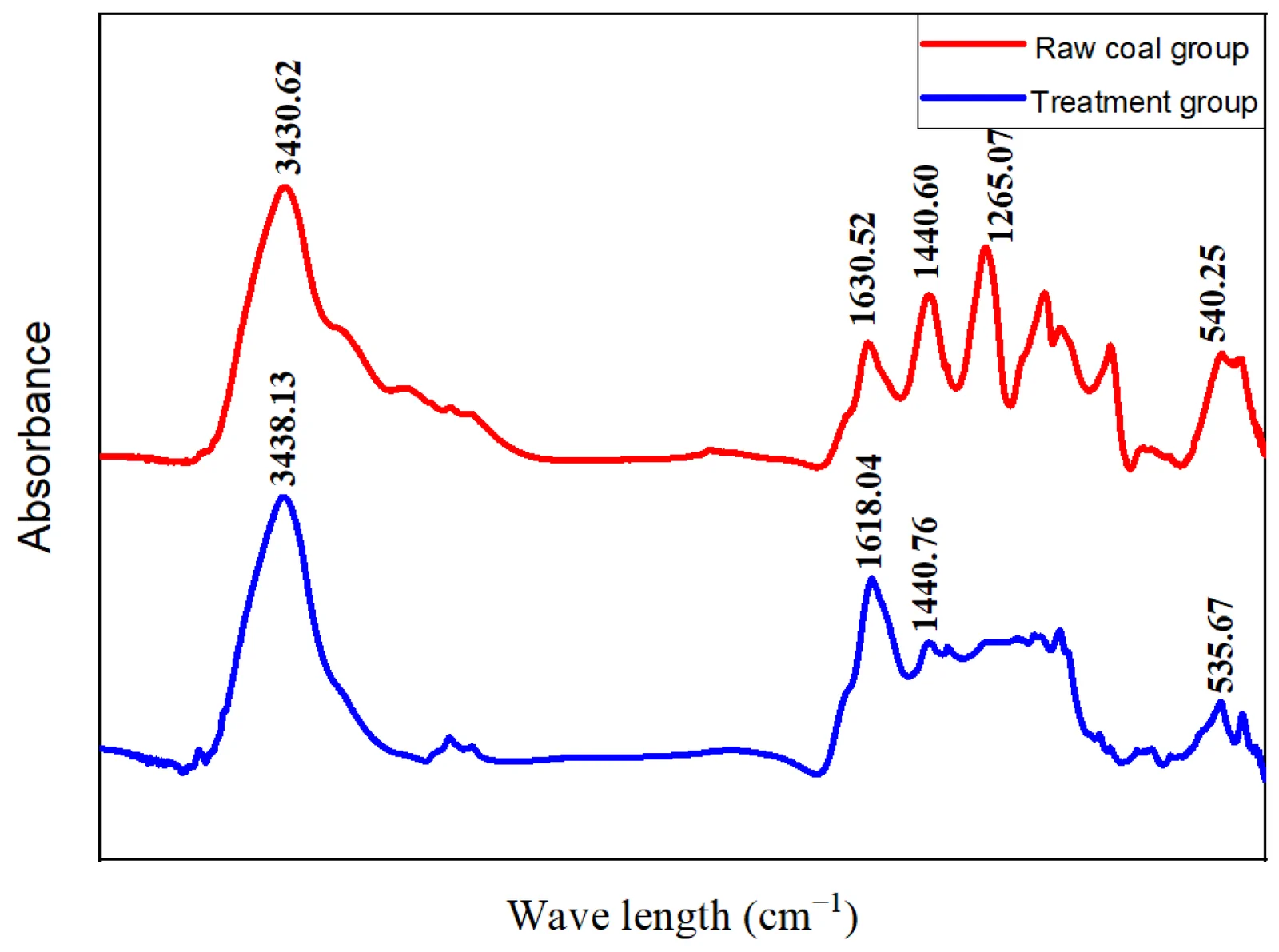
FTIR spectra of raw and suppressant-treated coal.

The spectra were smoothed using a Gaussian algorithm with 15 smoothing points. The resulting FTIR spectra are shown in Figure 13 [32].

The spectra of raw and treated coal were broadly similar, although the treated sample showed lower overall absorbance and several shifts in peak position and intensity. The O-H band shifted from 3438.13 cm^−1^ in raw coal to 3430.62 cm^−1^ after treatment, suggesting hydrogen bonding or related interactions between the suppressant and hydroxyl groups on the coal surface. The band at 1618.04 cm^−1^ shifted to 1630.52 cm^−1^, indicating a change in the local environment of carbonyl and/or aromatic C=C groups. Peak intensities between 1455 and 1000 cm^−1^ were also lower after treatment. In the low-wavenumber region, the band at 540.25 cm^−1^ shifted to 535.67 cm^−1^, providing further evidence of interactions between the suppressant and coal functional groups.

The spectra were deconvoluted in Origin. A second-derivative analysis was applied to each wavenumber region to determine the number of component peaks, which were then fitted with Gaussian functions. The fitting parameters were iteratively optimized by least squares until convergence [32].

**Figure 14:**
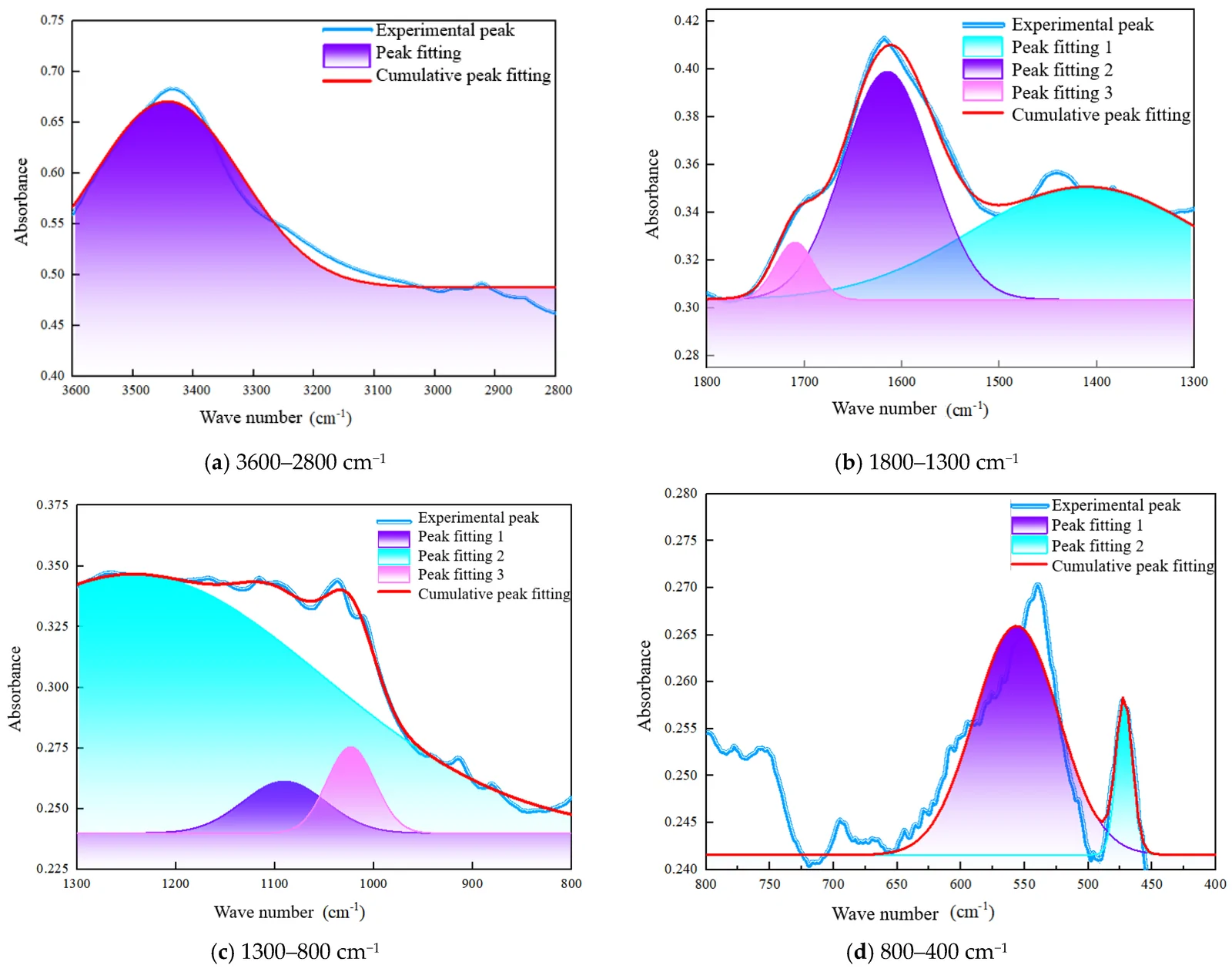
Curve-fitted FTIR spectra of raw coal in different wavenumber regions.

**Figure 15:**
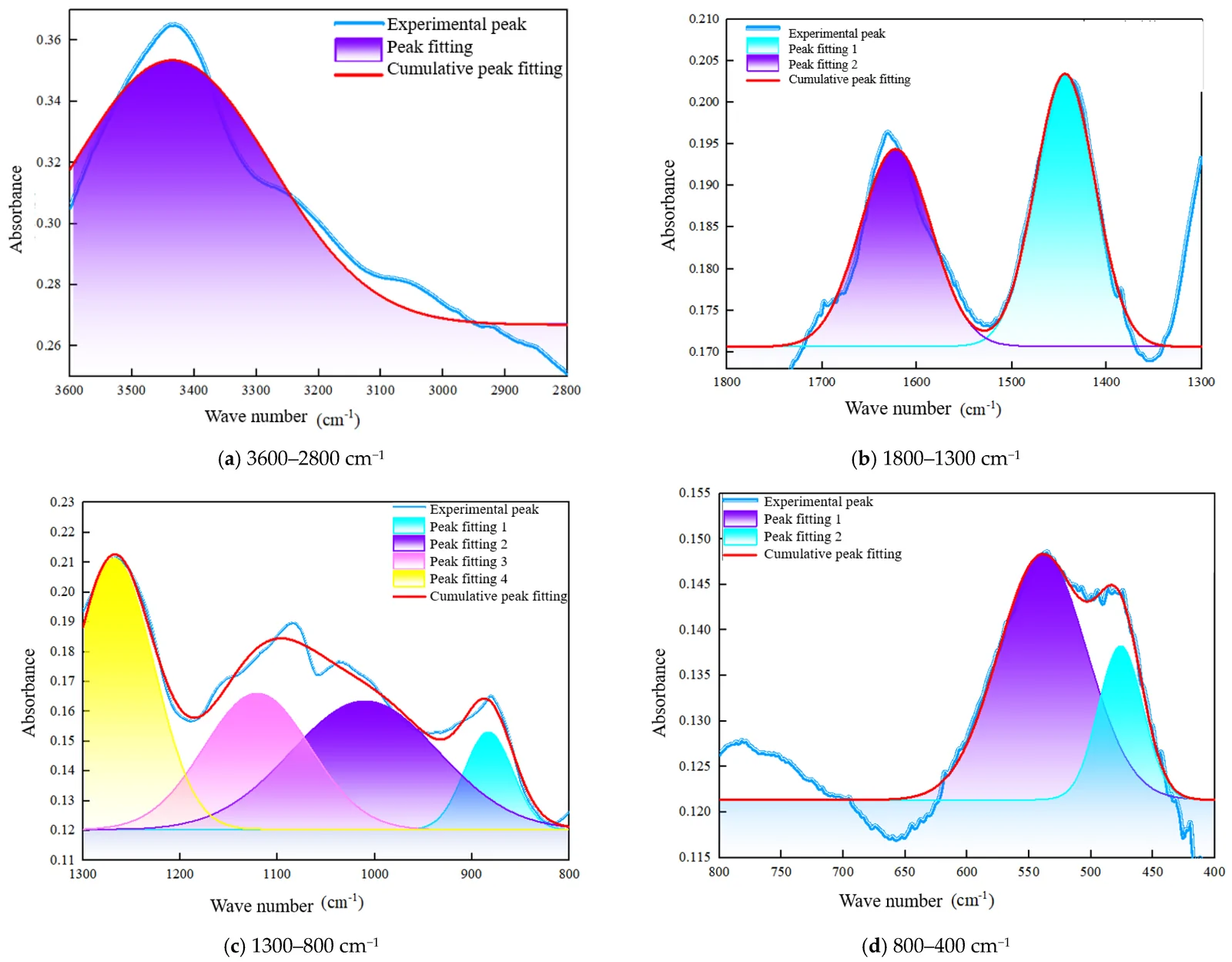
Curve-fitted FTIR spectra of suppressant-treated coal in different wavenumber regions.

The fitted component peaks of raw and suppressant-treated coal are shown in Figure 14 and Figure 15, respectively.

Raw coal exhibited a prominent band at 1618.04 cm^−1^, whereas treated coal showed a corresponding band at 1630.52 cm^−1^. These bands fall within the region associated with aromatic-ring C=C stretching. In the low-wavenumber region, raw coal showed bands at 540.25 and 473.31 cm^−1^, while treated coal showed bands at 880.61 and 535.67 cm^−1^; these bands were assigned to out-of-plane aromatic C-H bending. Neither sample showed a distinct band between 3110 and 3010 cm^−1^, indicating weak aromatic C-H stretching.

Thus, the principal aromatic features were ring C=C stretching and out-of-plane aromatic C-H bending.

Raw and treated coal showed CH_2_ antisymmetric stretching bands at 2920.53 and 2917.89 cm^−1^, respectively. Raw coal also showed a band at 2846.63 cm^−1^, assigned to symmetric CH_2_ stretching. Bands at 1440.76 cm^−1^ in raw coal and 1440.60 cm^−1^ in treated coal were attributed to asymmetric CH_3_ deformation. Bands near 1700 cm^−1^ were present in both samples and indicate changes in unsaturated and/or carbonyl-containing structures.

The main aliphatic features were therefore associated with CH_2_ stretching, CH_3_ deformation, and vibrations of unsaturated carbon-containing structures.

The broad O-H band shifted from 3438.13 cm^−1^ in raw coal to 3430.62 cm^−1^ after treatment and was assigned to alcohol and phenolic hydroxyl groups. Neither sample showed a distinct band between 3690 and 3620 cm^−1^, indicating a low concentration of free hydroxyl groups. The raw-coal band at 1701.17 cm^−1^ was assigned to carbonyl stretching in anhydrides, esters, and/or ketones. Bands at 1618.04 cm^−1^ in raw coal and 1630.52 cm^−1^ in treated coal were associated with carboxylate stretching. The band at 1264.89 cm^−1^ was attributed to carboxylic-acid and phenolic C-OH stretching. Bands at 1033.43 cm^−1^ in raw coal and 1028.61 cm^−1^ in treated coal were assigned to alcohol C-OH and ether C-O stretching.

Overall, the oxygen-containing groups in raw coal were represented primarily by hydroxyl, carbonyl, ether, and alcohol C-OH vibrations. After treatment, the dominant features remained hydroxyl, carboxylic-acid/phenolic C-OH, alcohol C-OH, and ether vibrations.

### 3.8. SEM Analysis

**Figure 16:**
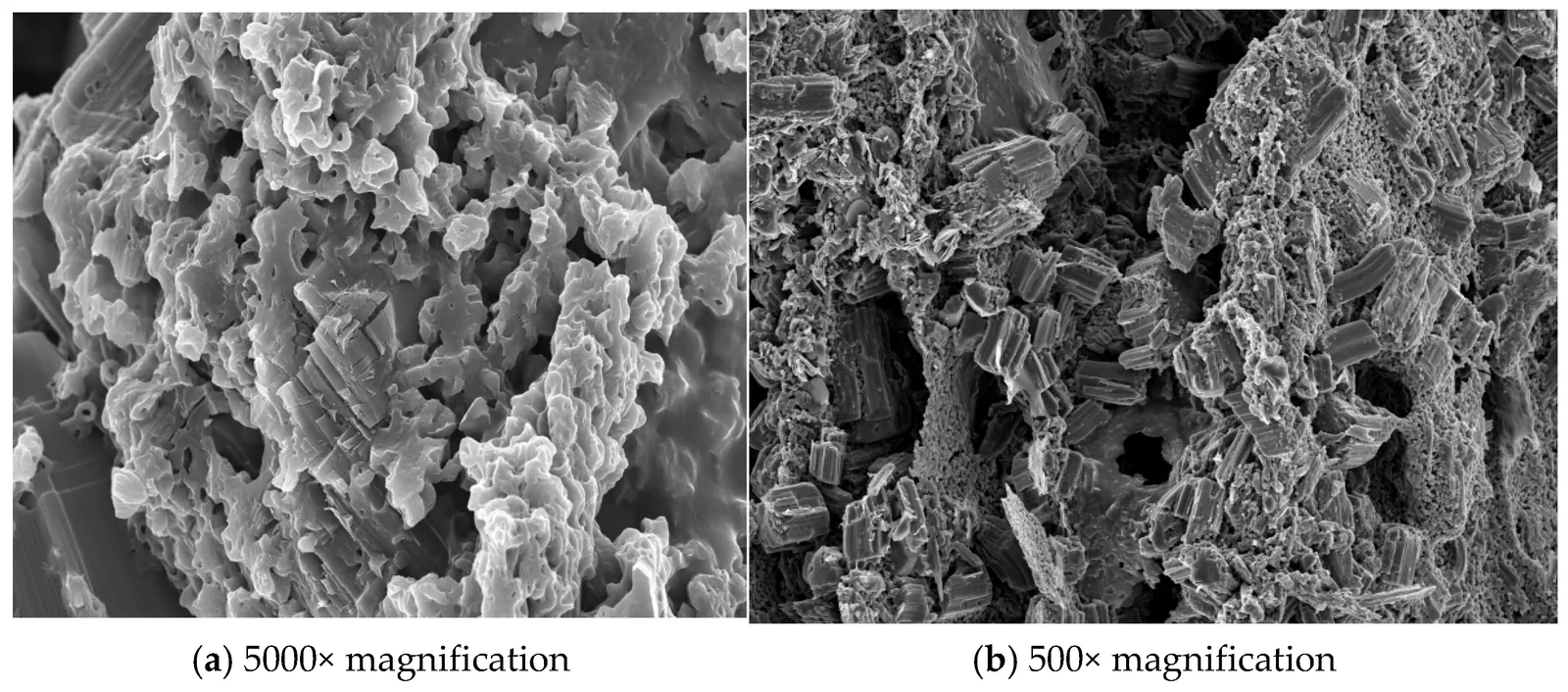
SEM images of the flame-retardant dust suppressant at different magnifications.

**Figure 17:**
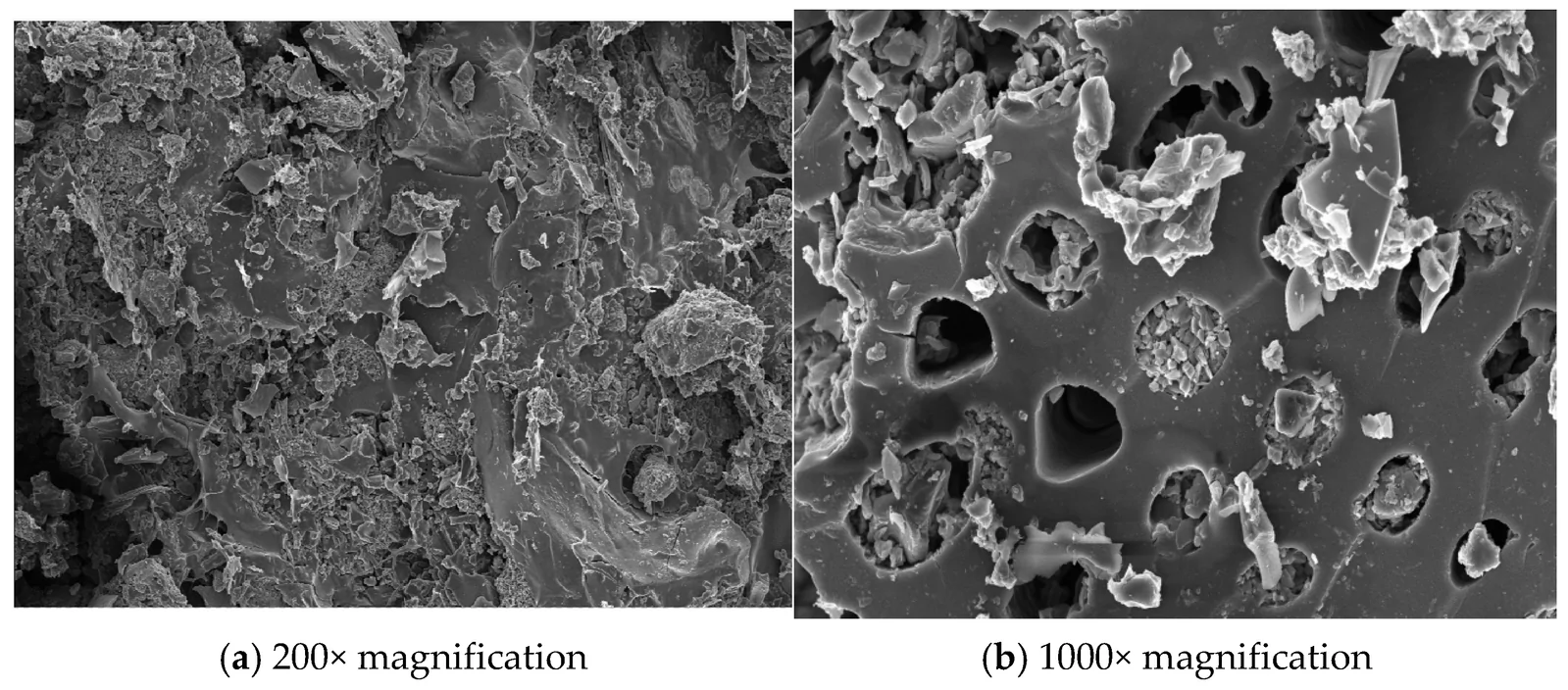
SEM images of suppressant-treated coal at different magnifications.

Figure 16 and Figure 17 show SEM images of the suppressant and suppressant-treated coal, respectively, at different magnifications.

The suppressant exhibited a loosely interwoven macromorphology containing block-like functional crystals within a porous matrix (Figure 16), a structure that favors spreading and adhesion on coal. At higher magnification, the matrix appeared as a compact, folded, flocculent network capable of forming a continuous surface film. The embedded crystals retain the active flame-retardant components, while nanoscale pores may enhance dust adsorption through capillary forces and maintain permeability.

The suppressant-treated coal was uniformly dispersed without obvious stratification or large agglomerates, indicating good compatibility between the suppressant and pulverized coal (Figure 17). The suppressant formed a continuous porous matrix in which fine coal particles adhered to the surface or occupied pores. At higher magnification, the particles were tightly embedded in the matrix or wrapped by its flocculent structure, with little evidence of particle detachment. These observations confirm that the film-forming matrix effectively coated and anchored the pulverized coal.

## 4. Conclusions

This study developed a CMC-based dual-function flame-retardant dust suppressant containing PCDI, APP, and ZB for integrated control of coal-dust dispersion and low-temperature coal oxidation. Orthogonal testing showed that PCDI and CMC were the principal variables affecting penetration depth, whereas APP and ZB had no significant effect within the investigated concentration ranges. Considering penetration, rheological behavior, and water-scour resistance, the formulation containing 1% CMC, 12% APP, 3.5% ZB, and 1.5% PCDI was selected as the optimal formulation.

The optimized suppressant demonstrated effective dust-control performance. Its stock solution achieved an average dust-suppression efficiency of 59.7% in the simulated-roadway test, markedly higher than that of water. Contact-angle measurements showed rapid spreading and penetration on coal surfaces, with the contact angle decreasing from 72.8° to 17.2° within 0.3 s. FTIR and SEM results further indicated that the suppressant interacted with coal-surface functional groups and formed a continuous porous crosslinked film that coated and anchored coal particles.

The suppressant also inhibited low-temperature coal oxidation. At 170 °C, the 10% treatment reduced CO release by 40.0%, while the apparent activation energy increased by an average of 29.73% relative to raw coal. These findings indicate that the material can simultaneously enhance dust consolidation and increase the energy barrier to coal oxidation.

Future research should focus on four specific directions to support industrial application: ① long-term stability assessment under simulated underground conditions, including resistance to repeated water scour, temperature fluctuation (−10~40 °C), and mechanical abrasion from mining equipment; ② compatibility evaluation with existing mine ventilation and water spraying systems, to optimize injection pressure and spraying parameters for full-scale roadway application; ③ environmental impact assessment, including biodegradability of CMC matrix, leaching toxicity of APP and ZB, and potential effects on mine water treatment systems; ④ large-scale field validation in working faces with different coal seam conditions (e.g., high-gas, high-spontaneous combustion risk mines) to verify the practical performance of the suppressant. These studies will provide critical data for the engineering promotion of the developed dual-function suppressant.

## Figures and Tables

**Table 1:** Main materials and reagents used in the experiments.

Material	Grade	Supplier
Carboxymethyl cellulose (CMC)	Analytical reagent (AR, 99%)	Sinopharm Chemical Reagent Co., Ltd. Shanghai, China
Ammonium polyphosphate (APP)	Analytical reagent (AR, 99%)	Ruiyuan Chemical Jinan, China
Zinc borate (ZB)	Analytical reagent (AR, 99%)	Tianjin Huasheng Chemical Reagent Co., Ltd. Tianjin, China
Polycarbodiimide (PCDI)	Analytical reagent (AR, 99%)	Guangzhou Wanjun Chemical Technology Co., Ltd. Guangzhou, China
Deionized water	Prepared in the laboratory	

**Table 2:** Orthogonal experimental design.

Run	CMC (%)	APP (%)	ZB (%)	PCDI (%)
1	1	8	2	0.5
2	1	12	3.5	1.5
3	1	16	5	2.5
4	1.5	8	5	1.5
5	1.5	12	2	2.5
6	1.5	16	3.5	0.5
7	2	8	3.5	2.5
8	2	12	5	0.5
9	2	16	2	1.5

**Table 3:** Characteristics of the coal sample used in the simulated-roadway test.

Coal Type	Analytical Indices				
Bituminous coal	M_ad_ (%)	A_d_ (%)	V_daf_ (%)	S_t,d_ (%)	V_d_ (cm^3^/g)
	3.21	3.32	33.12	0.33	0.82

**Table 4:** Measured penetration depths for the nine formulations.

Run	CMC (%)	APP (%)	ZB (%)	PCDI (%)	Penetration Depth (cm)
1	1	8	2	0.5	35.2
2	1	12	3.5	1.5	27.5
3	1	16	5	2.5	20.6
4	1.5	8	5	1.5	22.5
5	1.5	12	2	2.5	15.8
6	1.5	16	3.5	0.5	32.6
7	2	8	3.5	2.5	9.4
8	2	12	5	0.5	23.3
9	2	16	2	1.5	17.2

**Table 5:** Significance analysis of the effects of CMC, APP, ZB, and PCDI contents on penetration depth.

Factors	Sum of Squares	df	Mean Square	F	*p*
CMC	190.036	2	95.018	1.644	0.043
APP	2.842	2	1.421	0.016	0.384
ZB	1.616	2	0.808	0.009	0.591
PCDI	342.362	2	171.181	5.281	0.025

R^2^ = 0.956; *p* < 0.05; adjusted R^2^ = 0.932; predicted R^2^; Lack of Fit (F-value) = 2.14; Lack of Fit (*p*-value) = 0.172.

**Table 6:** Apparent activation energies of raw and treated coal in different temperature intervals.

Temperature Range (°C)	Apparent Activation Energy (kJ/mol)		
	Raw Coal	5% Treatment Group	10% Treatment Group
30–70	32.88	44.02	45.77
70–120	53.15	61.63	68.96
120–170	59.27	64.57	71.26

**Table 7:** Time-dependent contact angle and droplet-wetting state.

Time (s)	Contact Angle (°)	Range (°)	Droplet Volume (µL)	Wetting State
0	72.8 ± 2.1	70.7~74.9	10.89	Medium wetting
0.1	59.4 ± 1.9	55.5~61.3	8.07	Rapid wetting
0.2	41.1 ± 0.6	40.5~41.9	5.07	Efficient spreading
0.3	17.2 ± 1.5	15.7~19.7	1.82	Near-complete wetting

## Data Availability

The original contributions presented in this study are included in the article. Further inquiries can be directed to the corresponding author.
